# Inhibitory Effect of (2*S*)-Pinocembrin From *Goniothalamus macrophyllus* on the Prostaglandin E_2_ Production in Macrophage Cell Lines: *In Vitro* and *In Silico* Studies

**DOI:** 10.1155/2024/8811022

**Published:** 2024-10-30

**Authors:** Hilwan Yuda Teruna, Kamal Rullah, Rudi Hendra, Rahayu Utami, Deri Islami, Siti Munirah Mohd Faudzi, Mohd Fadhlizil Fasihi Mohd Aluwi, Kok Wai Lam

**Affiliations:** ^1^Department of Chemistry, Faculty Mathematics and Natural Sciences, Universitas Riau, Pekanbaru 28293, Indonesia; ^2^Department of Pharmaceutical Chemistry, Kulliyyah of Pharmacy, International Islamic University Malaysia 25200, Kuantan, Pahang, Malaysia; ^3^Department of Pharmacy, Sekolah Tinggi Ilmu Farmasi Riau, Pekanbaru 28293, Indonesia; ^4^Department of Pharmacy, Faculty of Medicine and Health Sciences, Universitas Abdurrab, Pekanbaru 28292, Indonesia; ^5^Natural Medicines and Product Research Laboratory (NaturMeds), Institute of Bioscience, Universiti Putra Malaysia, UPM Serdang, Selangor 43400, Malaysia; ^6^Faculty of Industrial Sciences and Technology, Universiti Malaysia Pahang, Gambang 26300, Pahang, Malaysia; ^7^Drugs and Herbal Research Centre, Faculty of Pharmacy, Universiti Kebangsaan Malaysia, Kuala Lumpur 50300, Malaysia

## Abstract

Pinocembrin (PCB), a flavonoid known for its anti-inflammatory properties, has been approved for various clinical trial applications. To evaluate deeper into the anti-inflammatory potential of the specific enantiomer of natural PCB, we conducted the first investigation into the efficacy of the pure enantiomer (2*S*)-PCB in modulating inflammatory mediators induced by lipopolysaccharide (LPS) in both murine RAW 264.7 and human U937 macrophage cell lines. This particular compound was isolated from *Goniothalamus macrophyllus* (Annonaceae), a native plant of Indonesia. This plant has been used traditionally as an herbal medicine to alleviate inflammation. (2*S*)-PCB was isolated from the stem bark of *G. macrophyllus* by defatting with *n*-hexane followed by maceration with methanol. Purification was performed using several chromatographic techniques. The absolute configuration was determined using electronic circular dichroism (ECD) spectroscopy. This compound was then tested for its inhibitory activity on prostaglandin E_2_ (PGE_2_) and subjected to docking simulations. The results indicated that (2*S*)-PCB significantly suppressed the production of PGE_2_ induced by LPS in both RAW 264.7 and U937 cell lines. The docking simulations revealed that (2*S*)-PCB reduced PGE_2_ levels by suppressing mitogen-activated protein kinase (MAPK) activation through inhibiting p38 and extracellular signal-regulated kinases (ERK). These findings suggest that the compound may prevent worsening of septic shock caused by bacterial infection.

## 1. Introduction

**Figure 1:**
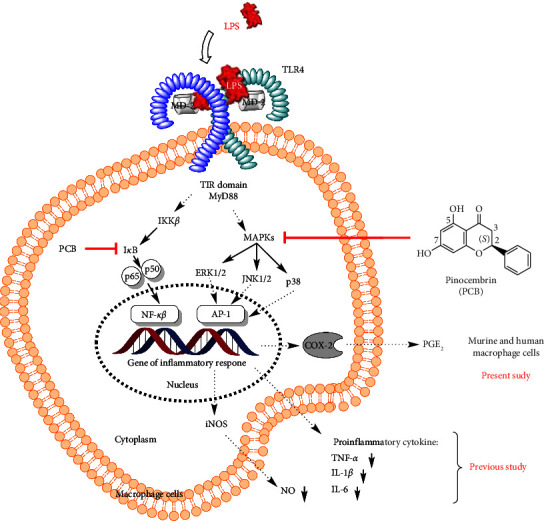
Schematic of the proposed molecular anti-inflammatory mechanism of (2*S*)-PCB in macrophages. The red lines indicate the targeted MAPKs.

Prostaglandin E_2_ (PGE_2_) is a significant product of arachidonic metabolism via the cyclooxygenase (COX) and prostaglandin E synthase pathways. However, it is well established that excessive production of PGE_2_ contributes to the development of inflammatory diseases such as atherosclerosis [1], angiogenesis [2, 3], rheumatoid arthritis [4, 5], and cancer [6–9] and pain [10]. Several studies have shown the conversion of arachidonic acid (AA) to prostaglandin H_2_ (PGH_2_) by the action of COX enzymes, specifically COX-1 and COX-2, followed by the transformation of PGH_2_ to PGE_2_ by microsomal prostaglandin E synthase-1/2 (mPGES-1/2) or cytosolic prostaglandin E synthase (cPGES). Subsequent reports have confirmed that cPGES and mPGES-2 are constitutively expressed in various tissues, whereas mPGES-1, like COX-2, is upregulated in response to several inflammatory stimuli, including lipopolysaccharide (LPS) [11–15]. Numerous studies have indicated that several inflammatory stimuli increase COX-2 expression via the activation of mitogen-activated protein kinases (MAPKs) [16, 17]. MAPKs are classified into four major groups: extracellular signal-regulated kinases 1 and 2 (ERK1 and ERK2), c-Jun *N*-terminal kinases (JNK1, JNK2, and JNK3), p38 kinases (p38*α*, p38*β*, p38*γ*, and p38*δ*), and ERK5/BMK1 kinases [18]. Upon exposure to prototypic inflammagen LPS, a crucial component of the outer membrane of Gram-negative bacteria, transcriptional regulation of COX-2 gene expression is excessively modulated by the MAPK family, whereas ERK and p38 kinase play a significant role in the signaling pathway for the stability of COX-2 mRNA (Figure 1) [19, 20].

Natural products (NPs) are rich sources of new drugs for treatments of emerging human diseases [21]. PCB (5,7-dihydroxyflavanone), a flavonoid derivative isolated from several plants, is effective as an anti-inflammatory agent [22–26]. This compound was approved as a new treatment for ischemic stroke by the China Food and Drug Administration and started for a Phase II clinical trial in 2008 [27–29]. On the other hand, Feng and colleagues demonstrated the therapeutic potential of the compound in a murine macrophage model of LPS-stimulated acute lung injury [30]. The compound inhibited TNF-*α*, IL-1*β*, and IL-6 production via attenuation of NF-*κ*B and MAPK activation, which includes I*κ*B*α*, ERK1/2, JNK, and p38 kinase. Recently, the same group reported the possibility of using PCB in a racemic form to prevent the onset of septic shock caused by LPS (Figure 1) [31]. However, despite a positive correlation with the abovementioned research, the anti-inflammatory effects of PGE_2_ on murine and human macrophage cells have not been reported previously. Additionally, recent reviews have indicated that the significance of racemic flavanone stereospecific disposition has gradually become apparent. Some innovative studies on the achiral pharmacodynamics and pharmacokinetics of pure enantiomers of PCB in their corresponding glycosides have been reported [32].

Our group has reported the anti-inflammatory properties of natural compounds and their derivatives associated with PGE_2_ for various applications in precious medicine over the last decade [33–36]. In our pursuit of novel anti-inflammatory agents, we have evaluated the effect of natural (2*S*)-PCB isolated from *Goniothalamus macrophyllus*, an Indonesian plant, on PGE_2_ production against the LPS-induced murine and human macrophage cell lines. Additionally, an *in silico* study was conducted to gain insight into the possible pathway involved in the PGE_2_ inhibitory activity of (2*S*)-PCB.

## 2. Materials and Methods

### 2.1. General Experimental Procedures

The UV-visible spectra were recorded using a Shimadzu UV-1800 spectrophotometer. Infrared (IR) spectra were recorded on a Shimadzu IR Affinity-1 FT-IR spectrophotometer fitted with a 1.5 round diamond crystal. The optical rotations were measured using a JASCO P-2000 polarimeter. ECD spectra were acquired using a JASCO J-810 spectropolarimeter with a path length of 0.1 cm and a concentration between 50 and 100 μM in methanol. 1D- and 2D-nuclear magnetic resonance (NMR) spectra were recorded on an Agilent 500 MHz NMR spectrometer with a 5 mm BBO probe. The experiments were performed in pyridine-*d*_5_ solvents using the residual solvent peaks as a reference for calibrating the obtained spectra. The MestReNova 6.0.2 software was used to analyze the NMR spectra. Chemical shifts are expressed in parts per million (ppm) and are given as *δ* values. The coupling constants (*J* values) are given in Hz and the multiplicities are abbreviated as follows: (*s*) singlet, (*d*) doublet, (dd) doublet of doublets, (ddd) doublet of doublet of doublets, (*t*) triplet, (dt) doublet of triplets, and (*m*) multiplet. Low-resolution electron ionization mass spectrometry (LREIMS) spectra were measured using a Hewlett Packard GC-MS (methyl silicone capillary column) with HP5970B mass selective detector operated in scanning mode (*m/z* 40–400). Data were processed with HP-ChemStation software. High-performance liquid chromatography (HPLC) analysis was performed on a Shimadzu (UFLC VP-ODS size 250 × 4.6 mm serial No. 3062669) and eluted with a gradient mixture of acetonitrile in water (40%–80%) at a flow rate of 1 mL/min.

### 2.2. Plant Material

The stem bark of *G. macrophyllus* was collected at Bukit Suligi, Riau Province, Indonesia, and was identified by the botanist Prof. Fitmawati at the Department of Biology, Faculty of Mathematics and Natural Sciences, Universitas Riau, Indonesia. A voucher specimen (117.a) was deposited in the herbarium of the Universitas Riau. The Confirmation Letter for this identification is no. 117.a/UN19.1.28/Bio/Botani/2014 on 12 April 2014.

### 2.3. Extraction and Isolation of the (2*S*)-PCB

The air-dried powdered stem bark of *G. macrophyllus* was pulverized using an herbal grinding machine. Approximately 1.7 kg of the powder was successively extracted with 4 L of *n*-hexane for 72 h at room temperature and subsequently with 4 L of methanol for 72 h at room temperature. The extracts were filtered through a sieve (150 μm), a cotton plug, and then filter paper. The filtrates were dried under a vacuum to obtain yellow-brown solid extract. The yield of the methanol extract obtained was 153.4 g (9.02%). A portion of the methanol extract (55 g) was fractionated by vacuum liquid chromatography (VLC) with a variety of eluent compositions, and six fractions were obtained using *n*-hexane (Merck, catalog number 1.04367)/ethyl acetate (Merck, catalog number 1.09623) as solvents (gradient 20%–100% ethyl acetate) and ethyl acetate (Merck, catalog number 1.09623)/methanol (Merck, catalog number 1.06009) as solvents (gradient of 20%–40% methanol). Silica gel GF_254_ (Merck, catalog number 1.07730) was used for this purpose. Finally, the third fraction was further purified by several times flash column chromatography (Merck, catalog number 1.09385) with *n*-hexane/ethyl acetate (gradient 20%–70% of ethyl acetate) to afford the compound and recrystallized in absolute ethanol (Merck, catalog number 1.00983). A crystalline solid was isolated (1.2 g or 2.1% yield with respect to plant material) and identified as PCB.

### 2.4. PGE_2_ Inhibitory Assay [34, 37]

#### 2.4.1. Cell Culture

The murine macrophage cell line (RAW 264.7) was obtained from the American Type Culture Collection (ATCC® TIB-71™, Manassas, United States of America) was maintained in Dulbecco's Modified Eagle's Medium (DMEM; ATCC® 30–2002™). The monocyte-like human lymphoma U937 cell line from the American Type Culture Collection (ATCC® CRL-1593.2™, Manassas, United States of America) was cultured in RPMI 1640 supplemented with DMEM (ATCC® 30–2001™). Subsequently, for differentiation into adherent macrophage-like cells, the U937 cell line (5 × 10^4^ cells/well) was incubated with 200 nM phorbol 12-myristate 13-acetate (PMA, Sigma-Aldrich) for 24 h, followed by washing twice with PBS pH 7.2, and then resting period of 24 h at 37°C in a humidified atmosphere with 5% CO_2._ Subsequently, the cells were used for stimulation and treatment.

#### 2.4.2. Cell Stimulation and Treatment

RAW 264.7 (5 × 10^4^ cells/well) and U937-PMA treated (5 × 10^4^ cells/well) cells were seeded into a tissue culture grade 96-well plate and incubated for 24 h at 37°C with 5% CO_2_. The attached cells were induced with a combination of 2 μg/mL LPS and 100 U/mL recombinant IFN-*γ* in the presence of (2*S*)-PCB at different concentrations (0–100 μM). Dimethyl sulfoxide (DMSO) was used as a solvent to add (2*S*)-PCB to the culture medium, and the final concentration of DMSO was 0.1% for all cultures. The control group was untreated with LPS/IFN-*γ*, and nimesulide was used as a positive control. Moreover, the LPS/IFN-*γ* treated cells serve as a baseline for comparison with experimental groups. The cells were then incubated at 37°C with 5% CO_2_ for 20 h. PGE_2_ levels were determined using a PGE_2_ Express EIA kit (Item No. 500141).

#### 2.4.3. Cell Viability

The viability of the isolated compound on cultured cells was determined by MTT (3-[4,5-dimethylthiazol-2-yl]-2,5 diphenyl tetrazolium bromide) assay. After treatment, the supernatant of the 96-well plate containing cells was removed and MTT reagent (5 mg/mL in PBS pH 7.2) was added to each well. The cells were incubated at 37°C with 5% CO_2_ for four hours, and the formazan salts were dissolved by adding 100 μL of DMSO. The absorbance was measured at 570 nm using a SpectraMax Plus microplate reader (Molecular Devices, Sunnyvale, California, United States of America).

#### 2.4.4. Determination of PGE_2_

Cell culture supernatants were collected and examined for PGE_2_ secretion using Express EIA kits (Cayman Chemical, Ann Arbor, Michigan, United States of America). Data were measured using a SpectraMax Plus microplate reader (Molecular Devices, Sunnyvale, California, United States of America). The concentration of PGE_2_ for each sample was calculated from their respective standard curves.

#### 2.4.5. Statistical Analysis

Statistical analysis was performed using one-way ANOVA test followed by Dunnett's multiple comparisons test. A one-tailed test value of *p* ≤ 0.05 was considered statistically significant. All dataset was analyzed using GraphPad Prism software version 7 (GraphPad Software, Inc., La Jolla, California, United States of America). The statistical tests were performed at the 95% confidence level.

### 2.5. Molecular Modeling [36]

#### 2.5.1. Computers and Software

All molecular modeling methods were performed using Discovery Studio® 3.1 (Accelrys, Inc., San Diego, California, United States of America) on an Intel® (TM)2 Quad CPU Q8200 @2.33 GHz running under a Windows XP Professional operating system. Some other molecular modeling software programs, including CHIMERA 1.9 and ChemDraw® Professional 15.0, were used in this study.

#### 2.5.2. Structure Preparation

The protein crystal structures were retrieved from the RCSB Protein Data Bank (PDB). The 2D structures of the ligand were built with ChemDraw® Professional 15.0 (PerkinElmer, Inc., Waltham, Massachusetts, United States of America). In brief, the ligands were prepared using the modules implemented in Discovery Studio® 3.1, such as removing duplicates, enumerating isomers and tautomers, and generating 3D conformations. Several protocols require reasonable starting ligand structures to achieve good results. The protocols may also benefit from enumerating valid ionization states, tautomers, and isomers (e.g., the docking protocols). The protocol accomplishes this by performing the following steps, some of which can be controlled by the protocol parameters, including generating canonical tautomer, keeping only the largest fragments, setting standard formal charges on common functional groups, kekulizing molecules, enumerating ionization states at a given pH range or setting them according to predefined templates, enumerating tautomers, enumerating isomers, removing duplicate structures, and fixing bad valences. In the next step, the minimised energy of the synthesised ligand incorporated various attributes, including the initial potential energy (kcal/mol, the molecule's energy prior to minimisation), root mean square (RMS) gradient (kcal/mol × Å, the concluding RMS gradient of the minimised molecule), and CHARMM energy (kcal/mol, the final energy of the minimised molecule).

#### 2.5.3. Molecular Docking

Molecular docking studies were performed on the crystal structure of subfamily proteins of MAPKs, including p38*α* JNK, and ERK, by using the CDOCKER protocol under the receptor-ligand interaction section in Discovery Studio® 3.1 (Accelrys, San Diego, United States of America). All the protein crystal structures of the inhibitor-bound MAPKs were retrieved from the Brookhaven PDB IDs: p38*α* (**1A9U**, 2.50 Å), JNK1 (**3V3V**, 2.70 Å), JNK2 (**3NPC,** 2.35 Å), and ERK2 (**5BVD**, 1.90 Å). In this study, the calculation docking protocol was performed using cDOCKER, which is a grid-based molecular docking tool utilized by CHARMM forcefield. The CDOCKER score is expressed as a negative number derived from the hydrogen bonds, van der Waals forces, and electrostatic interactions between the target protein and the ligand. The lowest value indicates a more favorable binding into the active site. The top ten ligand-binding poses were ranked according to their CDOCKER energies, and the predicted binding interactions were analyzed. All the proteins were pretreated by hydrogen atoms, and all ionizable residues were set at their default protonation states at a neutral pH of 7.4. The ligand was heated to a temperature of 700 K in 2000 steps and cooled by 300 K in 5000 steps. A sphere shape was generated for the active site as grid points for the atomic coordinate of the docking study. The grid extension was set to range 10–15 Å.

#### 2.5.4. Docking Validation

The CDOCKER protocol was validated via redocked experiments to ensure the accuracy of the docking program. Each original ligand of protein p38*α*, JNK, and ERK was redocked into the receptor's coordinated active site to precisely reproduce the orientation and position of the protein-ligand observed in the crystal structure. The top-ranking conformational clusters from this dock were evaluated by the root mean square deviation (RMSD) value of the difference between redocked and original ligand poses. The low RMSD (below 2.0 Å) was acceptable for the molecular docking method.

## 3. Results and Discussion

### 3.1. Isolation and Elucidation Structure of (2*S*)-PCB

The natural PCB was isolated from *G. macrophyllus* as yellowish crystal needles. The purity of PCB was analyzed by HPLC and this confirmed that the compound was pure. LREIMS analysis indicated peaks at *m/z* 256 (M^+^). The complete NMR spectra (1D and 2D), including ^1^H, ^13^C, COSY, HMQC, and HMBC, were used in elucidating the PCB structure. Their proton and carbon assignments are compared to published data.

The optical activity of the isolated compound showed a specific rotation value of [*α*]_D_^22^ – 22.0° (c 1.67 mg/mL, DMSO), and the absolute configuration was measured by comparative ECD spectroscopy. The FT-IR spectrum showed some functional groups that support the chemical structure of (2*S*)-PCB.

The ^1^H NMR spectroscopy of the compound depicted aliphatic resonances at *δ* 5.55 (dd, *J* = 13.0; 3.0 Hz), 2.97 (dd, *J* = 17.0; 3.0 Hz), and 3.21 (dd, *J* = 17.0 Hz; 13.0 Hz) from the methine and methylene protons (H-2 and H-3, see the PCB structure in Figure 1), respectively, indicating the presence of a flavanone. Through extensive NMR analyses, the compound was identified as PCB, and its spectroscopy data were compared to PCB isolated by Ching et al. [38]. Additionally, COSY spectra analysis revealed the proton–proton correlation between H-2 and H-3, and the coupling constants of those protons indicated that the position of the ring B in the flavanone was considered as *S* configuration. PCB was optically active with a specific rotation value of [*α*]_D_^22^ – 22.0° (c 1.67 mg/mL, DMSO), and the absolute configuration was determined using comparative ECD spectroscopy with a positive cotton effect observed at 325 nm (Δ*ε* 115.00), indicating that it was in the *S* configuration, in agreement with previously published data [38–43]. As a result, the isolated compound was designated as (2*S*)-PCB.

### 3.2. PGE_2_ Production Assay

**Figure 2:**
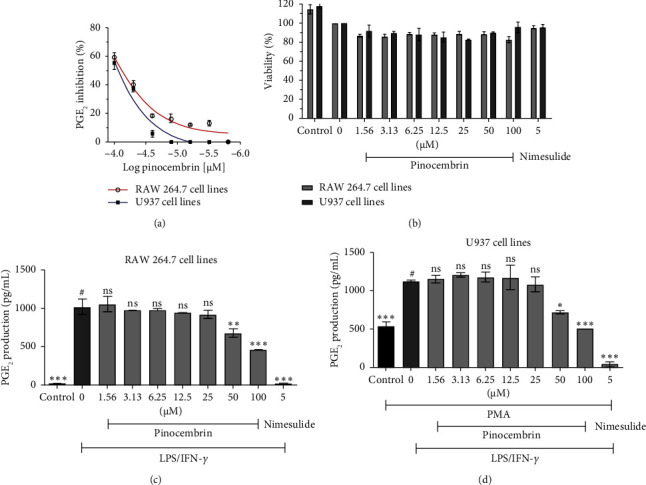
Dose–response curve of PCB on PGE_2_ inhibition in LPS/IFN-*γ* stimulated RAW 264.7 and U937 cells (a). Effect of (2*S*)-PCB on RAW 264.7 and U937 cell viability (b). The concentration effect of PCB ranges from 1.56 to 100 μM in RAW 264.7 (c) and U937 (d) cells. The values are expressed as means ± SD of dual individual samples. ⁣^∗^*p* <  0.05, ⁣^∗∗^*p*  <  0.01, ⁣^∗∗∗^*p* <  0.001, indicating significant differences from LPS/IFN-γ–treated cells as group # (ns denotes nonsignificance, with nimesulide serving as the reference chemical for PGE2 inhibition).

Figure 2 depicts the inhibition of PGE_2_ production by (2*S*)-PCB isolated from *G. macrophyllus*. The figure shows the dose–response relationship of PCB on PGE_2_ inhibition in RAW 264.7 and U937 cells stimulated with LPS/IFN-*γ*.

In general, natural (2*S*)-PCB isolated from *G. macrophyllus* inhibited murine RAW 264.7 macrophage cell lines with IC_50_ of 75.9 μM, whereas it inhibited human U937 macrophage cell lines with IC_50_ of 86.4 μM (Figure 2(a)). This is consistent with a previous report in which (2*S*)-PCB was shown to have a moderate inhibitory effect on PGE_2_ production in macrophage cells [44]. The results are acceptable when the inhibition was not affected by viability in both cells at the tested concentration (Figure 2(b)). As a result, the compound inhibition of PGE_2_ production cannot be equated with cell cytotoxicity. The dose-dependent graphs of PCB on the inhibition of PGE_2_ in RAW 264.7 and U937 macrophages are shown in Figures 2(c) and 2(d).

### 3.3. Molecular Modeling

**Figure 3:**
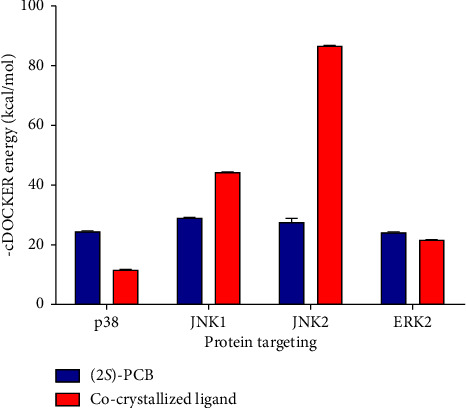
Comparison of CDOCKER interaction energies of active compounds compared with the co-crystallized inhibitors in p38*α*, JNK1, JNK2, and ERK. The values are presented as mean ± SD based on ten ligand-binding poses, assessed by their CDOCKER energy. Lower scores and values indicate stronger binding affinities.

Molecular docking studies of the compound against PGE_2_ production were performed on the crystal structures of subfamily proteins of MAPKs by using the CDOCKER protocol. A comparison of CDOCKER interaction energies of the compound compared to the co-crystallized inhibitors in p38*α*, JNK1, JNK2, and ERK2 is shown in Figure 3.

**Figure 4:**
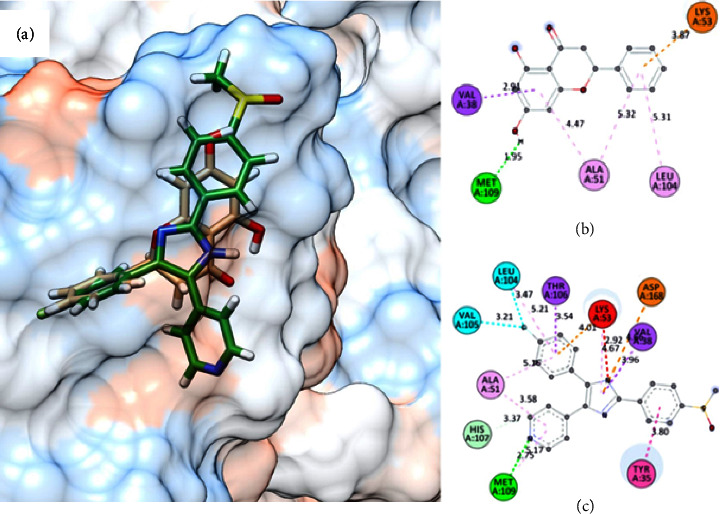
Overlay of the docked conformation of co-crystallized ligand SB203580 and PCB (a). The ligand SB203580 is green in color based on carbon atom while PCB is brown color based on the atom. Plausible important binding interactions of PCB (b) and SB203580 (c) with the binding residues of p38*α* ATP-binding site as depicted in the 2D diagram.

Among the target enzymes, (2*S*)-PCB exhibited a favorable CDOCKER interaction energy of −24.4 kcal/mol (p38*α*) and −24.1 kcal/mol (ERK2), which closely resembled the CDOCKER interaction energy of their co-crystallized ligand, −11.4 kcal/mol and −21.5 kcal/mol, respectively. In the p38*α* binding site, the hydroxyl group of (2*S*)-PCB could be interacted by forming a strong hydrogen bond with Met190 (1.95 Å) and a weak *π*-*π* bond (3.87 Å) with Lys553 (Figure 4).

Previous studies demonstrated that the racemic form of PCB inhibited LPS-induced PGE_2_ production and significantly inhibited TNF-*α*, IL-1*β*, and IL-6 production by disrupting MAPK activation involving p38, JNK, and ERK kinases [30]. As a consequence, the focus on the *S* configuration of PCB was employed in the docking simulation on the human protein crystal structure of p38*α* (**1A9U**), JNK1 (**3V3V**), JNK2 (**3NPC**), and ERK2 (**5BVD**). The CDOCKER interaction energy was used to compare the binding affinity of the compound with co-crystallized ligands (see Figure 3).

According to the results, the compounds favored binding to p38*α* with an average binding interaction energy of −24.4 kcal/mol, higher than the co-crystallized p38*α* inhibitor, **SB203580**, which has binding interaction energy of −11.4 kcal/mol. The hydroxyl group could interact with Met190 (1.95 Å) *via* a strong hydrogen bond and with Lys553 via a weak *π*-*π* bond (3.87 Å) (Figure 4). Interestingly, these interactions were also observed in **SB203580**, the co-crystallized ligand of p38*α*, where the imidazole rings of **SB203580** form 2.75 Å and 4.01 Å hydrogen bonds with Met109 and Lys53, respectively. This in-depth examination of (2S)-PCB revealed that it might be a similarly effective act to attenuate PGE_2_*via* blocking p38*α* with **SB203580**. In general, (2*S*)-PCB binds to selected enzyme targets with a lower affinity than their co-crystallized ligands, despite their potent bioactivity. The previous report corroborated these findings, demonstrating that (2*S*)-PCB reduced PGE_2_ production by inhibiting MAPK activation via suppressing p38 and ERK phosphorylation [44]. Regardless of the computational study used, the hit compound (2S)-PCB must be experimentally evaluated, including methods such as western blotting and gene expression analysis, to verify p38 and ERK as direct molecular targets and confirm prediction accuracy of the formation of (2S)-PCB/p38 or ERK complexes.

### 3.4. Docking Validation

In validating the docking protocol, it is essential that the RMSD between the redocked original ligand and the cocrystallized original ligand is below 2.0 Å. This threshold is assessed by superimposing the redocked and cocrystallised structures, then measuring their deviation. This requisite must be fulfilled for all four proteins examined in our research: p38*α*, JNK1, JNK2, and ERK2, in order for the investigation to continue. From the CDOCKER docking protocol, the original ligands were docked accurately with an RMSD value below 2.0 Å, which is considered an acceptable docking method. The top-ranked RMSD values for validating all four proteins with CDOCKER docking methods were recorded.

Additionally, pose accuracy in benchmarking studies showed similar proportions of the accurate pose. It was observed that the top-ranked ligand poses of the redocked original ligand closely resembled those of the cocrystallized original ligand: complex compound SB203580-p38*α* MAP kinase (PDB ID 1A9U).

Given the acknowledged potential of (2*S*)-PCB as an anti-inflammatory agent, it is proposed that this compound holds promise for development as an anti-inflammatory treatment targeting a wide array of inflammatory conditions. These conditions span from those caused by microbial infections (including bacteria, viruses, and fungi), to those induced by physical agents such as burns, stress, trauma from cuts, and radiation exposure, as well as those triggered by chemical agents like drugs, toxins, and alcohol. Additionally, (2*S*)-PCB may potentially alleviate inflammation stemming from immunologic reactions, such as those seen in rheumatoid arthritis. However, it is imperative to recognize potential limitations and challenges in the development and application of PCB-based therapeutics, including issues related to bioavailability, metabolic stability, possible side effects, and the need for further elucidation of its mechanisms of action in various inflammatory contexts. Moreover, the variability in response among individuals and the complexity of inflammatory pathways may hinder its widespread efficacy. These challenges become a focus or an opportunity for those interested in pursuing them. Thus, while (2*S*)-PCB shows promise, rigorous preclinical and clinical studies are warranted to fully assess its safety, efficacy, and limitations as an anti-inflammatory agent across diverse inflammatory conditions.

## 4. Conclusions

The stereoselective natural form of (2*S*)-PCB was successfully isolated from the stem bark of *G. macrophyllus*, marking the first instance of its extraction from this botanical source. Remarkably, upon evaluation, (2*S*)-PCB exhibited notable anti-inflammatory properties by effectively suppressing the production of PGE_2_ in both murine RAW 264.7 and human U937 macrophage cell lines. Building upon these observations, we postulate that the underlying mechanism driving this anti-inflammatory activity of (2*S*)-PCB involves inhibiting pivotal signaling pathways, particularly p38 and ERK, as a comprehensive docking study suggested.

These significant findings underscore the potential of natural (2*S*)-PCB as a novel therapeutic candidate in preventing septic shock, a severe condition often triggered by bacterial infections. By targeting key inflammatory mediators and pathways implicated in the pathogenesis of septic shock, (2*S*)-PCB holds promise as a potential intervention to mitigate the onset and progression of this life-threatening complication. This discovery highlights the pharmacological significance of (2*S*)-PCB and underscores the importance of exploring natural compounds derived from botanical sources as potential therapeutics for combating inflammatory diseases and related complications. Further investigations into the precise mechanisms of action and therapeutic efficacy of (2*S*)-PCB are warranted to validate its clinical potential and pave the way for its development as a novel anti-inflammatory agent.

## Data Availability

The data used to support the findings of this study are included in the supporting information file.

## Supporting Information

Figure S1 shows LREIMS of the PCB molecular ion (M^+^) peak, recorded at *m*/*z* 256. This is consistent with the molecular formula of PCB, C_15_H_12_O_4_. Mass spectrometry was recorded on Hewlett Packard GC-MS (methyl silicone capillary column) with HP5970B mass selective detector operated in scanning mode (*m*/*z* 40–400). Data were obtained from HP-ChemStation software.

The complete NMR spectra (1D and 2D) can be seen in Figures S2 for ^1^H-NMR, S3 for ^13^C-NMR, S4 for HMQC, S5 for COSY, and S6 for HMBC. These methine and methylene protons were typical signals of H-2 and H-3 from a flavanone (Figure S2). Seven aromatic signals are also determined, two protons of typical H6 and H8 signals from ring A, and five protons of H2′, H3′, H4′, H5′, and H6′ signals from ring B of a flavonoid. The protons' integrations show that there were 11 protons with one broad methoxy proton. Figure S3 shows the 15 carbon signals. A direct correlation between each proton and its carbon is described in Figure S4. The correlation of protons to protons in their spin systems is shown in Figure S5. Figure S6 shows how the carbons and protons are correlated in two to four bonds in the skeleton. Hence, the position of each carbon could be determined. The proton and carbon assignments of PCB are described in Table S1. Although the proton chemical shifts of PCB are slightly different from those of PCB reported by Ching et al. [38], their carbon chemical shifts are the same. The NMR solvent used in our research was pyridine-d5. Instead, Ching et al. used methanol-d4. This is why their proton chemical shifts look different.

The purity of PCB was analyzed by HPLC, and the chromatogram is shown in Figure S7. This confirmed that the compound was pure. Figure S8 shows the UV (a) and ECD (b) spectra of PCB. It is obvious that the UV maxima of PCB are similar to those of a flavanone. The absolute configuration was determined using comparative ECD spectroscopy, with a positive cotton effect observed at 325 nm (Δ*ε*115.00). This confirmed that the PCB is in S configuration. ECD spectra were obtained using Jasco CD (J-815 model), at 20°C, in 10 mm cell, and scanning speed of 50 nm/min.

The FT-IR spectrum showed some functional groups that support the chemical structure of (2*S*)-PCB. Figure S9 shows some vibrations. Vibrations of O-H, aromatic C-H, aliphatic C-H, and ketone C=O are observed at 3060 cm^−1^, 2910 cm^−1^, and 1595 cm^−1^, respectively. Again, this supports the functional group of a flavanone.

The top-ranked RMSD values for validating all four proteins with the CDOCKER docking method are listed in Table S2. Figure S10 shows the superimposed top-ranked ligand pose of the redocked original ligand and the cocrystallized original ligand.

Figure S11 shows extraction and isolation diagram of PCB.
