# Supplementary material for: Inhibitory Effect of (2S)-Pinocembrin From Goniothalamus macrophyllus on the Prostaglandin E2 Production in Macrophage Cell Lines: In Vitro and In Silico Studies
Source: Adv Pharmacol Pharm Sci. 2024 Oct 30;2024:8811022. doi: 10.1155/2024/8811022 (PMC11540893; doi:10.1155/2024/8811022)
Supplement: Supporting Information — Additional supporting information can be found online in the Supporting Information section. [file 8811022.f1.pdf]

SUPPLEMENTARY DATA

The inhibitory effect of (2*S*)-pinocembrin from *Goniothalamus macrophyllus* on the PGE_2 production in murine and human macrophage cell lines and *in silico* investigation on MAPK pathway

Hilwan Yuda Teruna[a, *], Kamal Rullah[b, c, *], Rudi Hendra[a], Deri Islami[d], Rahayu Utami[c], Siti Munirah Mohd Faudzi[e], Mohd Fadhlizil Fasihi Mohd Aluwi[f], Lam Kok Wai[g]

[a]*Department of Chemistry, FMIPA-Universitas Riau, Kampus Bina Widya Km 12.5, Pekanbaru, 28293, Indonesia.*

[b]*Department of Pharmaceutical Chemistry, Kulliyyah of Pharmacy, International Islamic University Malaysia, Bandar Indera Mahkota, 25200 Kuantan, Pahang, Malaysia.*

[c]*Sekolah Tinggi Ilmu Farmasi Riau, Jalan Kamboja, Pekanbaru, 28293, Indonesia.*

[d]*Program Studi S1 Farmasi, Faculty of Medicine and Health Sciences, Universitas Abdurrab, Jalan Riau Ujung, Pekanbaru, 28292, Indonesia.*

[e]*Natural Medicines and Product Research Laboratory (NaturMeds), Institute of Bioscience, Universiti Putra Malaysia, 43400 UPM Serdang, Selangor, Malaysia.*

[f]*Faculty of Industrial Sciences & Technology, Universiti Malaysia Pahang, Lebuhraya Tun Razak, 26300 Gambang, Pahang, Malaysia.*

[g]*Drugs and Herbal Research Centre, Faculty of Pharmacy, Universiti Kebangsaan Malaysia, Jalan Raja Muda Abdul Aziz, 50300 Kuala Lumpur, Malaysia.*

A. FIGURES

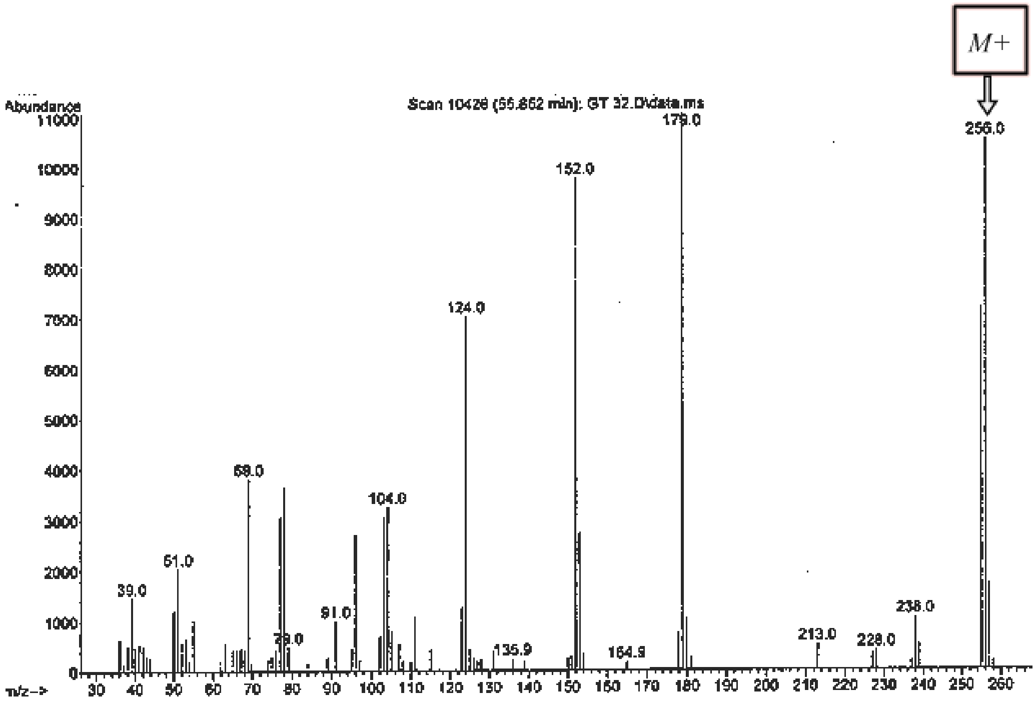

Figure S1. LR-EIMS of (2*S*)-pinocembrin (Hewlett Packard GC-MS (methyl silicone capillary column) with HP5970B mass selective detector operated in scanning mode (*m/z* 40-400). Data was obtained from HP-ChemStation Software.)

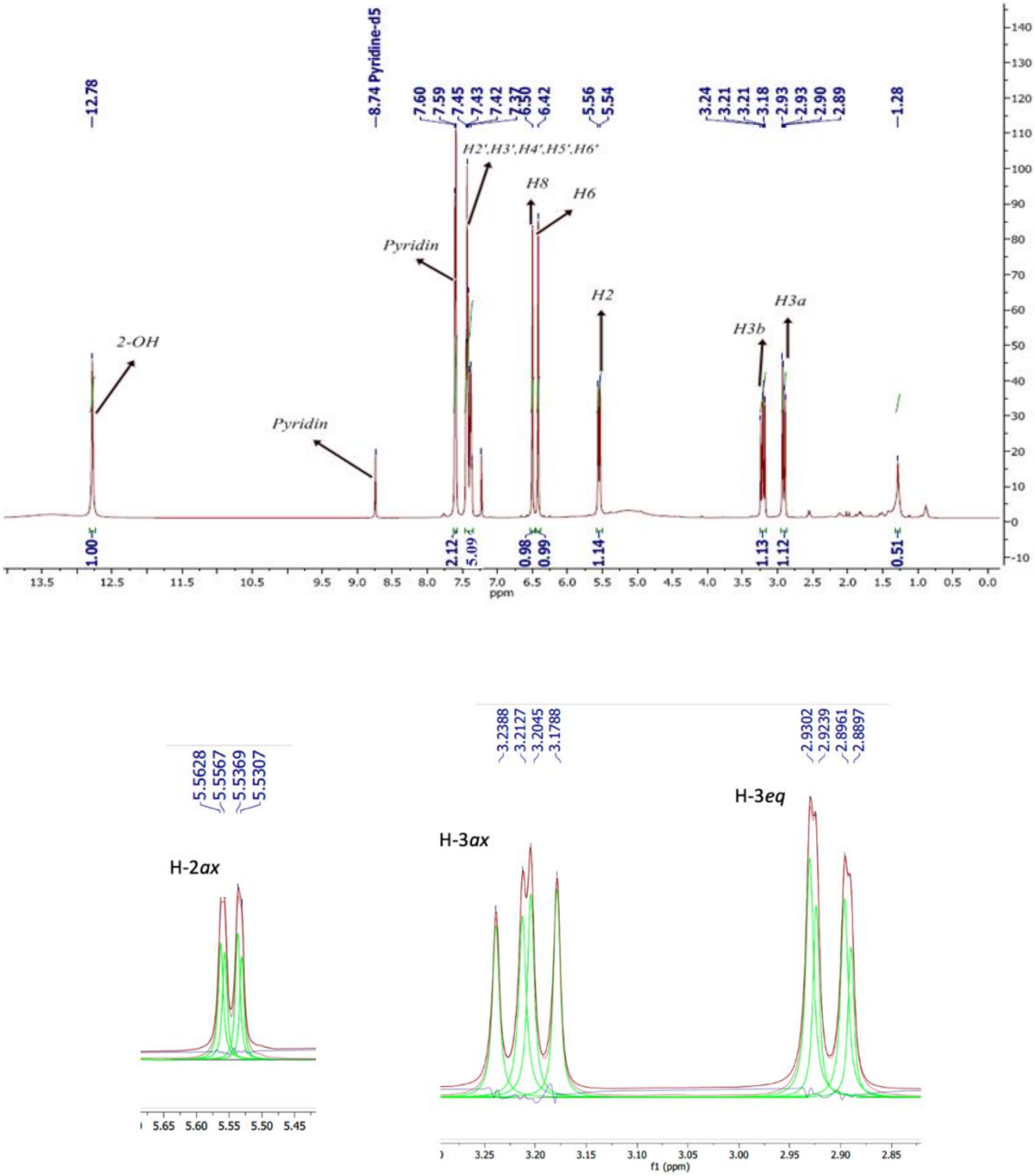

Figure S2. ^{1}H-NMR spectrum of (2*S*)-pinocembrin (Agilent 500 MHz NMR spectrometer with a 5 mm BBO probe; pyridine-*d*5 as a solvent)

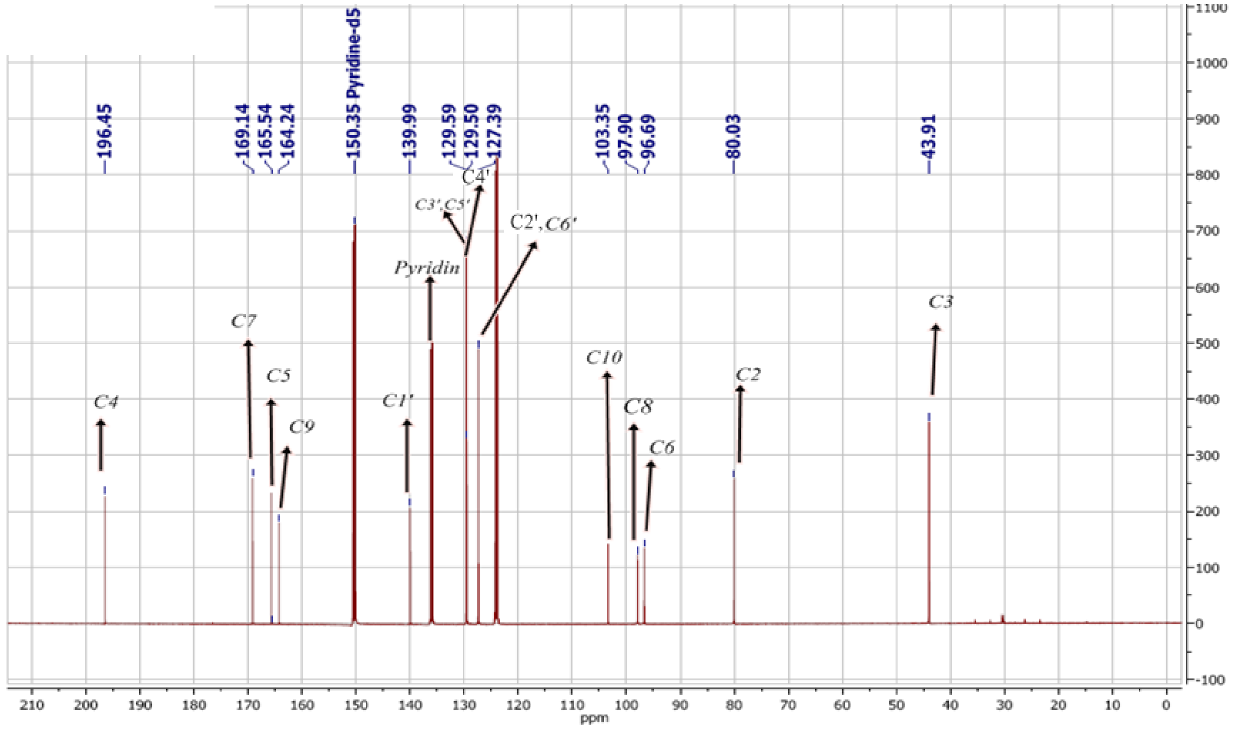

Figure S3. ^{13}C-NMR spectrum of (2*S*)-pinocembrin

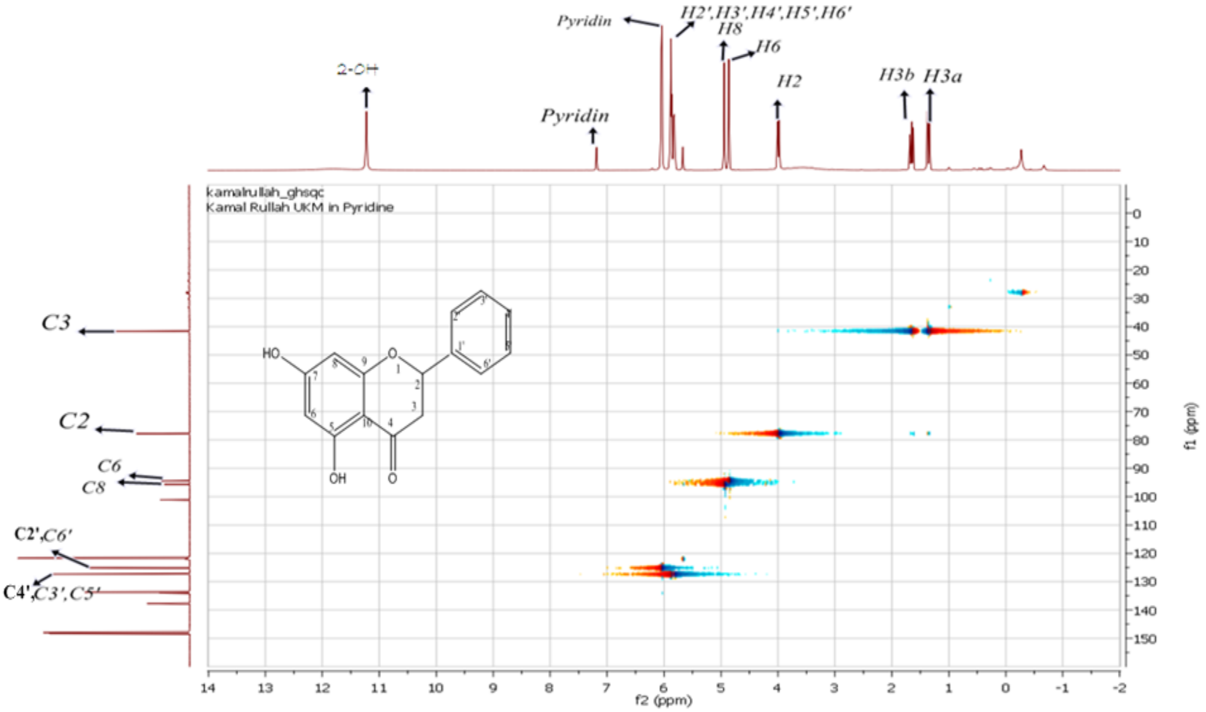

Figure S4. HMQC of (2*S*)-pinocembrin

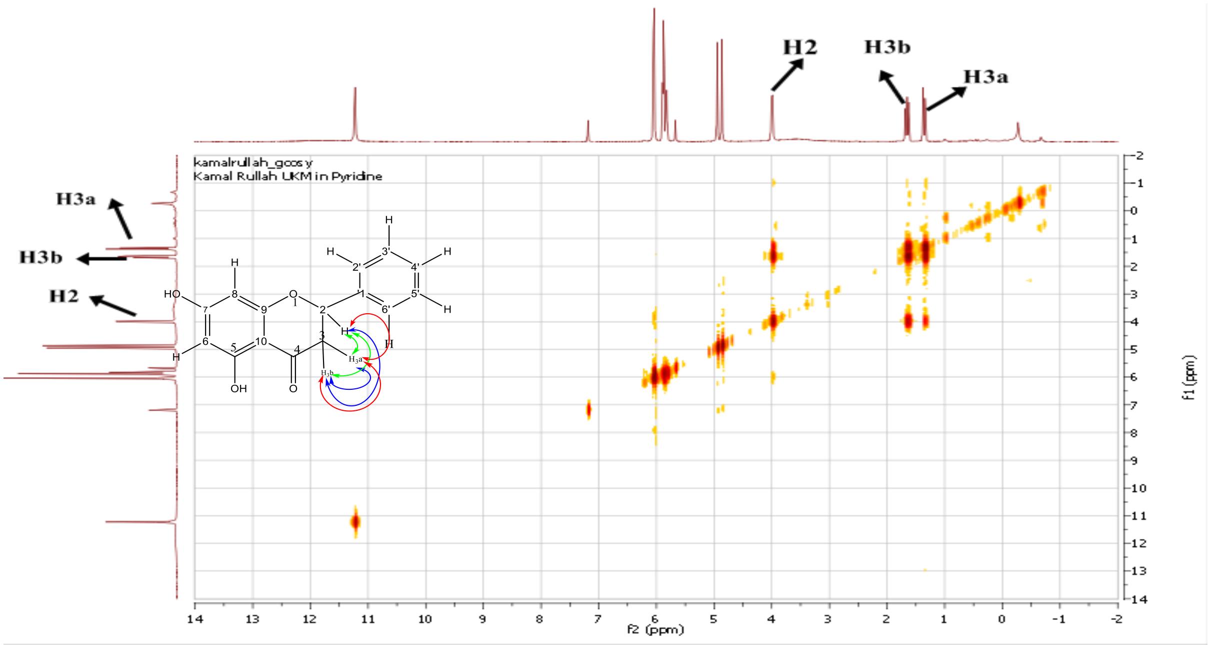

Figure S5. COSY of (2*S*)-pinocembrin

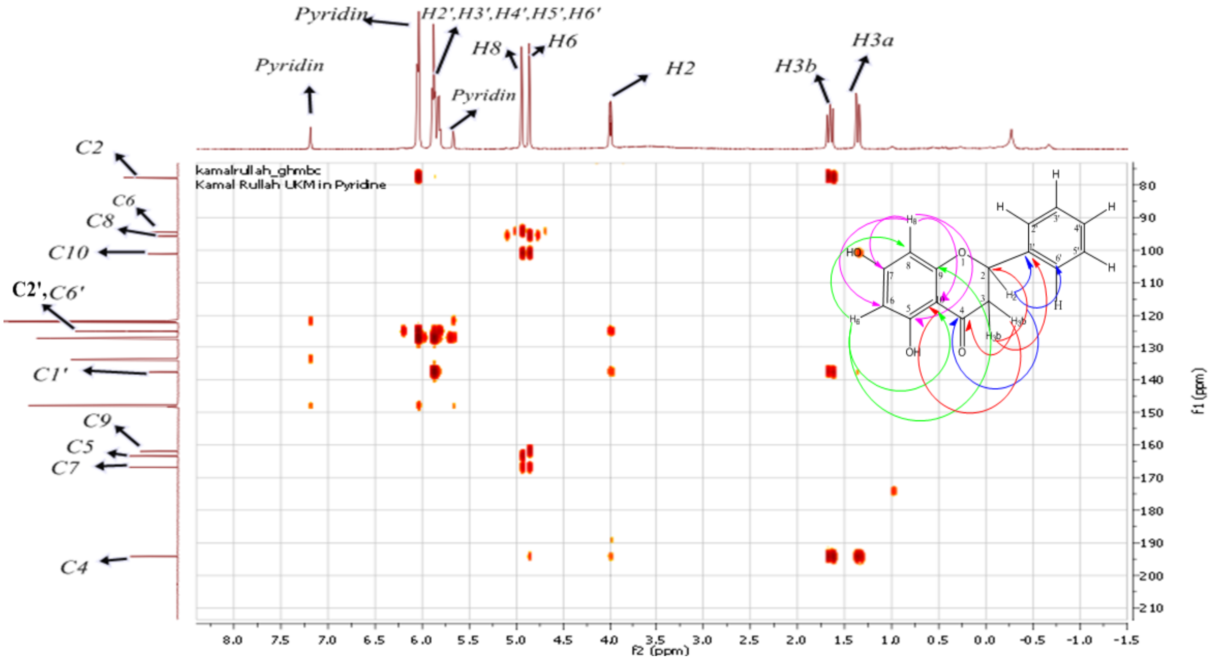

Figure S6. HMBC of (2*S*)-pinocembrin

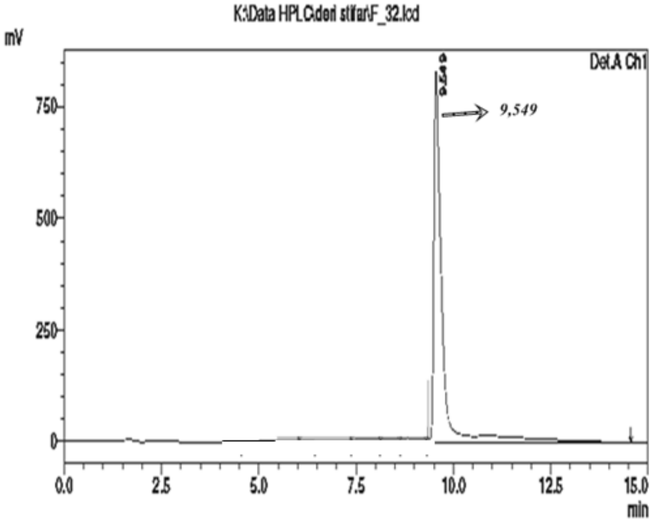

Figure S7. HPLC chromatogram of isolated (2*S*)-pinocembrin. The HPLC analysis for (2S) PCB was recorded with Shimadzu LC System with Shim-pack VP-ODS, 150 mm (length) x 4,6 (internal diameter) and 5 μm size. Gradient elution was employed (40% to 80% acetonitrile in water) with the flow rate of 1 mL per minute.

(a)

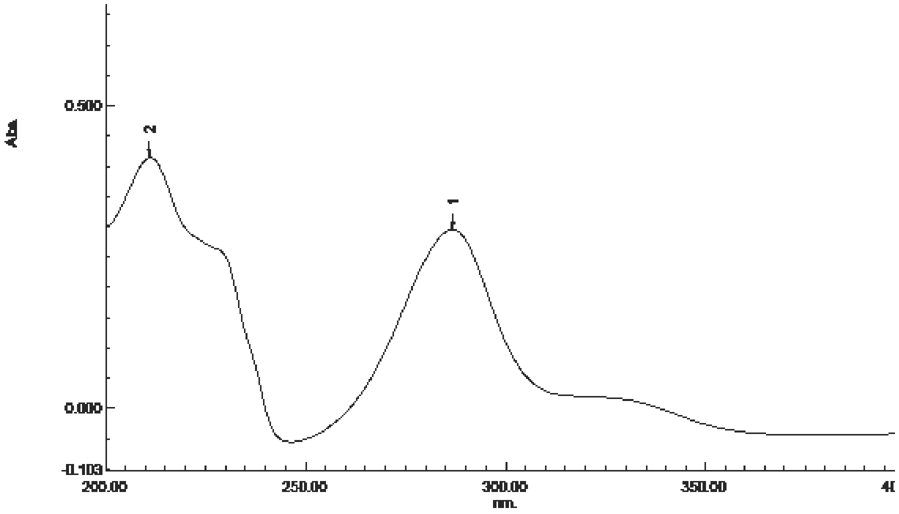

(b)

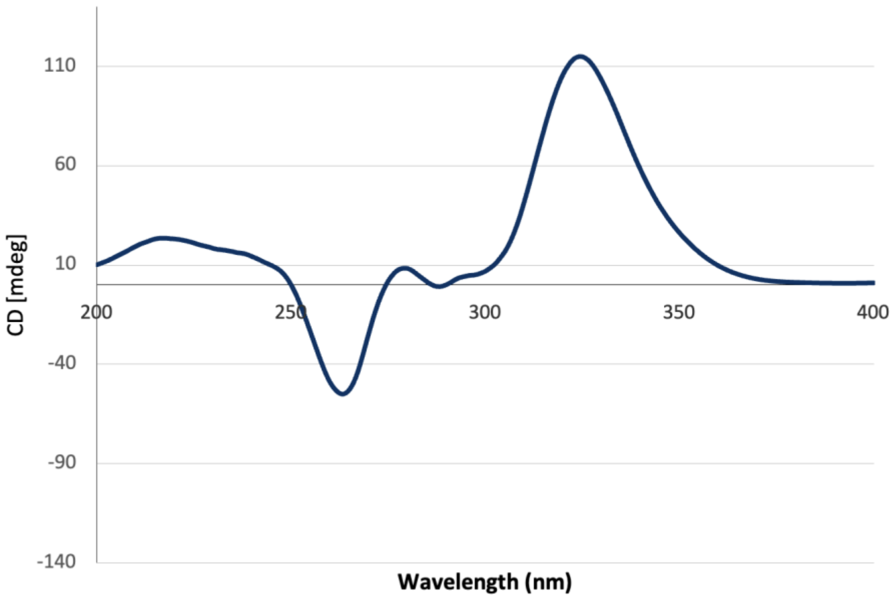

Figure S8. UV (a) and ECD (b) spectra of (2S)-pinocembrin (Jasco CD (J-815 model), at 20 ºC, in 10 mm cell, and scanning speed 50 nm/min.)

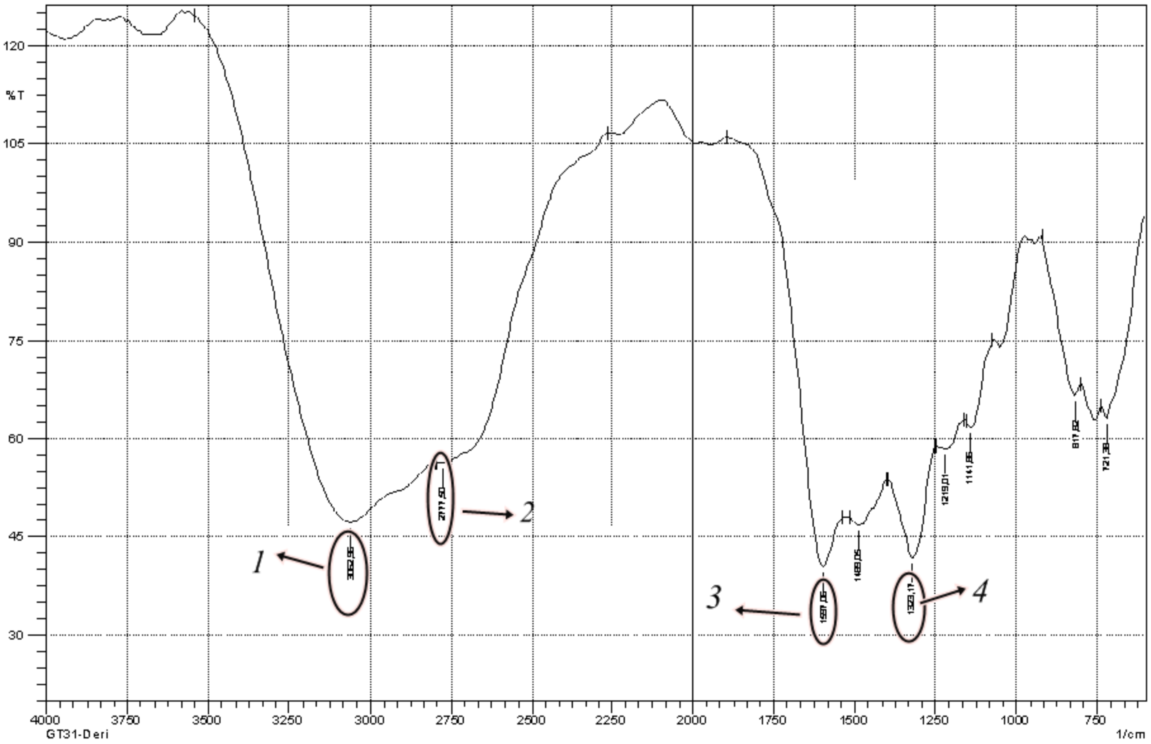

Figure S9. FTIR spectrum of (2*S*)-pinocembrin. Infrared (IR) spectra were recorded on a Shimadzu IR Affinity-1 FT-IR spectrometer fitted with a 1.5 round diamond crystal.

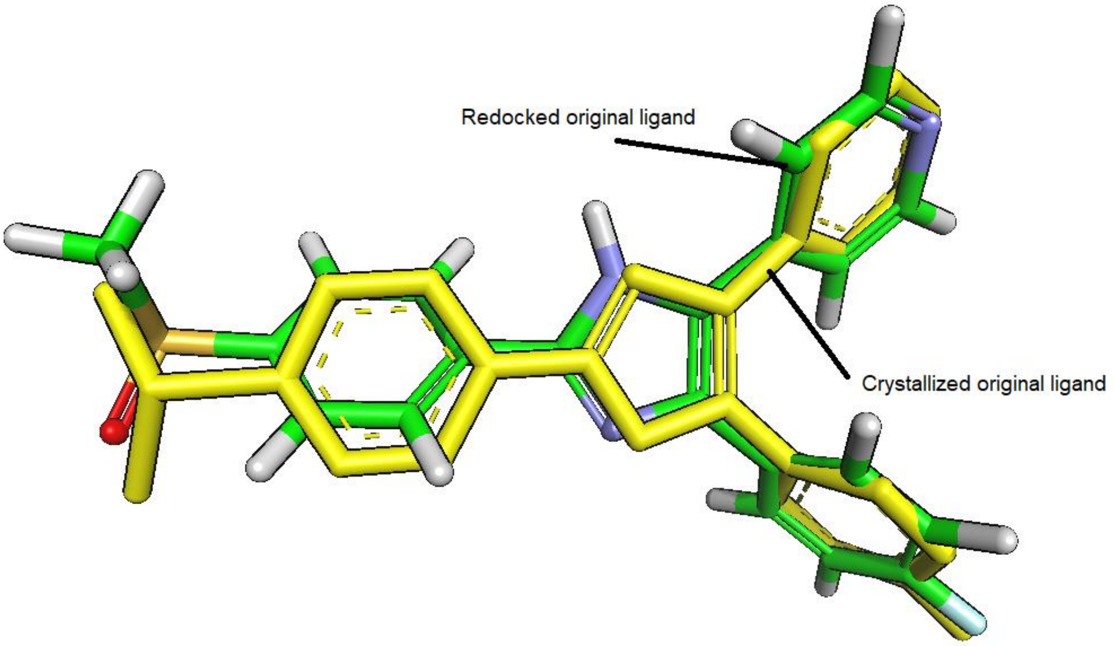

Figure S10. The top-ranked ligand pose for the redocked original ligand (green) compound SB203580- p38α MAP kinase (PDB ID 1A9U) superimposed with the co-crystallized original ligand (yellow)

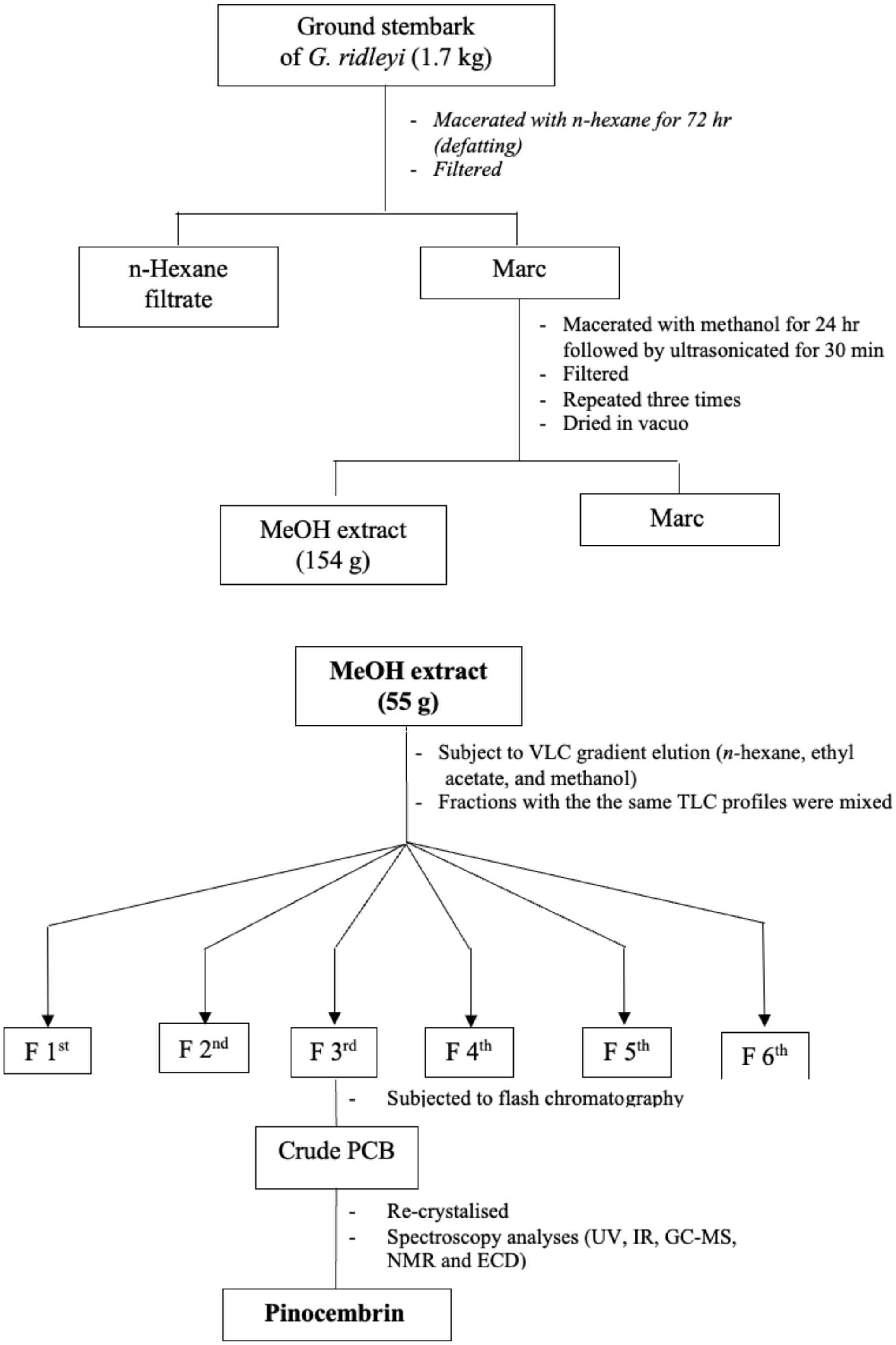

Figure S11. Extraction and isolation diagrams of PCB.

TABLES

Table S1. 1D-NMR spectral data of (2*S*)-pinocembrin

	Isolated compound		Pinocembrin† (Ching et al. 2007)	
**Position**	**δ_H (*J* in Hz)**	**Carbon δ_C**	**δ_H (*J* in Hz)**	**Carbon δ_C**
2	5.55 (dd, 13 Hz, 3 Hz)	80.0	5.33 (dd, 12.84, 3.68)	80.4
3	2.92 (dd,17 Hz, 3 Hz)<br>3.21 (dd, 17 Hz, 13 Hz)	43.9	2.67 (dd, 16.96, 3.68)<br>2.98 (dd, 17.40, 12.88)	44.1
4		196.5		197.3
5	12.78 (OH)	165.5	12.00 (OH)	165.4
6	6.42 (s)	97.9	5.87	96.2
7		169.1		168.3
8	6.50(s)	96.8	5.87	97.2
8a		164.24		164.6
4a		103.4		103.3
1'		140.0		140,3
2'	7.41 (m)	127.4	7.34	127.4
3'	7.41 (m)	129.6	7.34	129.6
4'	7.41 (m)	129.5	7.34	129.7
5'	7.41 (m)	129.6	7.34	129.6
6'	7.41 (m)	127.4	7.34	127.4

† Recorded on JEOL FT-NMR Spectrometer at 400 MHz (^{1}H) and 100 MHz (^{13}C) in CD_3OD.

Table S2. The top-ranked RMSD values for the validation of docking methods using cDOCKER

Protein	PDB ID	RMSD
p38α	1A9U	1.0678
JNK1	3V3V	0.7711
JNK2	3NPC	0.3303
ERK2	5BVD	0.7537
